# Modified Technique of Single-Bone Forearm in the Treatment of Deformities

**DOI:** 10.7759/cureus.26361

**Published:** 2022-06-27

**Authors:** Hamza Benameur, Souhail Bensaleh, Najib Alidrissi, Abdeloihab Jaafar, Mohammed Chahbouni

**Affiliations:** 1 Orthopaedics and Traumatology, Cheikh Khalifa International University Hospital, Mohammed VI University of Health Sciences (UM6SS), Casablanca, MAR

**Keywords:** one bone forearm, single bone forearm, salvage procedure, forearm surgery, hereditary multiple exostoses, limb deformity

## Abstract

The single-bone forearm is a salvage technique for massive loss of bone due to serious trauma, malignant tumors, infections or congenital deformity. It is also described to treat the sequelae of hereditary multiple exostoses disease that affects the distal end of the ulna. We present the case of a 29-year-old patient, operated for sequelae of hereditary multiple exostoses disease of the left forearm by a modified single-bone forearm technique. The patient, right-handed, operated on twice in childhood for a hereditary multiple exostoses disease of the left forearm: incomplete excision of the exostosis of the distal end of the ulna and lengthening of this last on external fixator, without improvement. The patient presented for a deformation of the left forearm with shortening compared to the right side‌. Significant limitation of prono-supination (pronation 15°, supination 20°). Elbow flexion at 110° and extension with deficit of 15°. Wrist flexion at 50° and extension at 50°, radial inclination at 25° and ulnar at 30°. The pain score was 3 according to the Visual Analogue Scale (VAS), especially on effort. Dash score was 31,82/100. We chose the forearm technique with a single bone. The immediate postoperative result found a realignment of the forearm, without neurological or vascular damages. Consolidation was obtained in four months. At five months, the patient recovered elbow flexion at 110° and full extension, wrist flexion at 45° and extension at 50°. Radial inclination at 20° and ulnar at 25°. The single-bone forearm technique has been described, not only for the treatment of hereditary multiple exostoses disease, but also for serious trauma or tumors with massive loss of bone. The technique generally consists of an osteotomy of the radius as well as the ulna, fixing the radius to the ulna creating a synostosis, with or without resection of part of one or both bones of the forearm. The most described complications of single-bone forearm procedure are pain, complications related to soft tissue secondary to the previous injury, and infections. The one-bone forearm remain a salvage technique for massive loss of bone of the forearm, or large deformities due to congenital malformations. This technique could allow the excision of massive bone and keep only a part of the ulna and the radius, with function maintenance and aesthetic forearm preservation.

## Introduction

The single-bone forearm is a salvage technique for massive loss of bone due to serious trauma, malignant tumors [1], infections or congenital deformity [2,3]. It is also described to treat the sequelae of hereditary multiple exostoses disease that affects the distal end of the ulna. This last one is a relatively rare entity, causing deformation of the forearm in children with limitation of pronation and supination [4]. Its adequate pediatric surgical management allows the patient to recover the form and function of the forearm. However, an exostosis of the forearm in children that goes unnoticed or is poorly treated leaves sequelae that are badly tolerated in adults [5].

We present the case of a patient operated on for sequelae of hereditary multiple exostoses disease of the left forearm by a modified single-bone forearm technique.

## Case presentation

**Figure 1:**
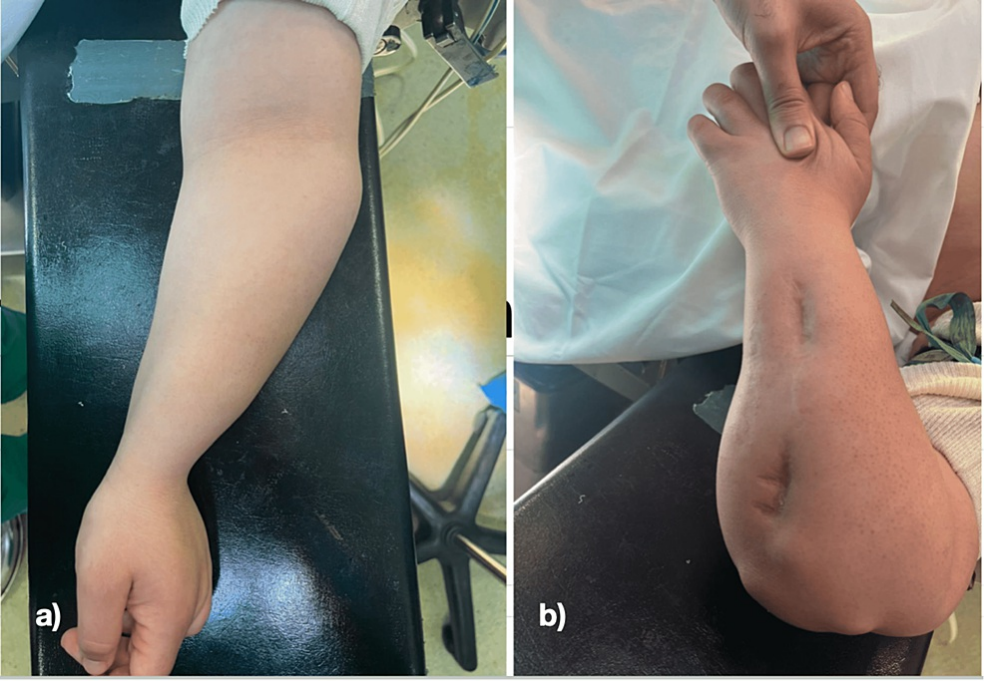
a) Deformation of the forearm before single bone forearm procedure. b) Scars of external fixator for ulna lengthening, in a previous surgery in childhood.

A 29-year-old woman, right-handed, operated on twice in childhood for a hereditary multiple exostoses disease of the left forearm with an incomplete excision of the exostosis of the ulna’s distal end and lengthening of this last on external fixator, without improvement. The patient presented for a deformation of the left forearm with shortening compared to the right side, associated to two invaginated surgical scars with adhesions on the ulnar shaft (Figure 1).

The radial head was palpable and protruding on the external aspect of the elbow. A significant limitation of the prono-supination was noticed, with a pronation limited to 15°, and a supination limited to 20°. The elbow flexion was limited to 110° and the extension to 15°. The wrist flexion and extension were both limited to 50°. The radial and ulnar inclinations were respectively limited to 25° and 30°. The pain score was at three according to the Visual Analogue Scale (VAS), especially on effort. Additionally, the Dash score was at 31,82/100.

**Figure 2:**
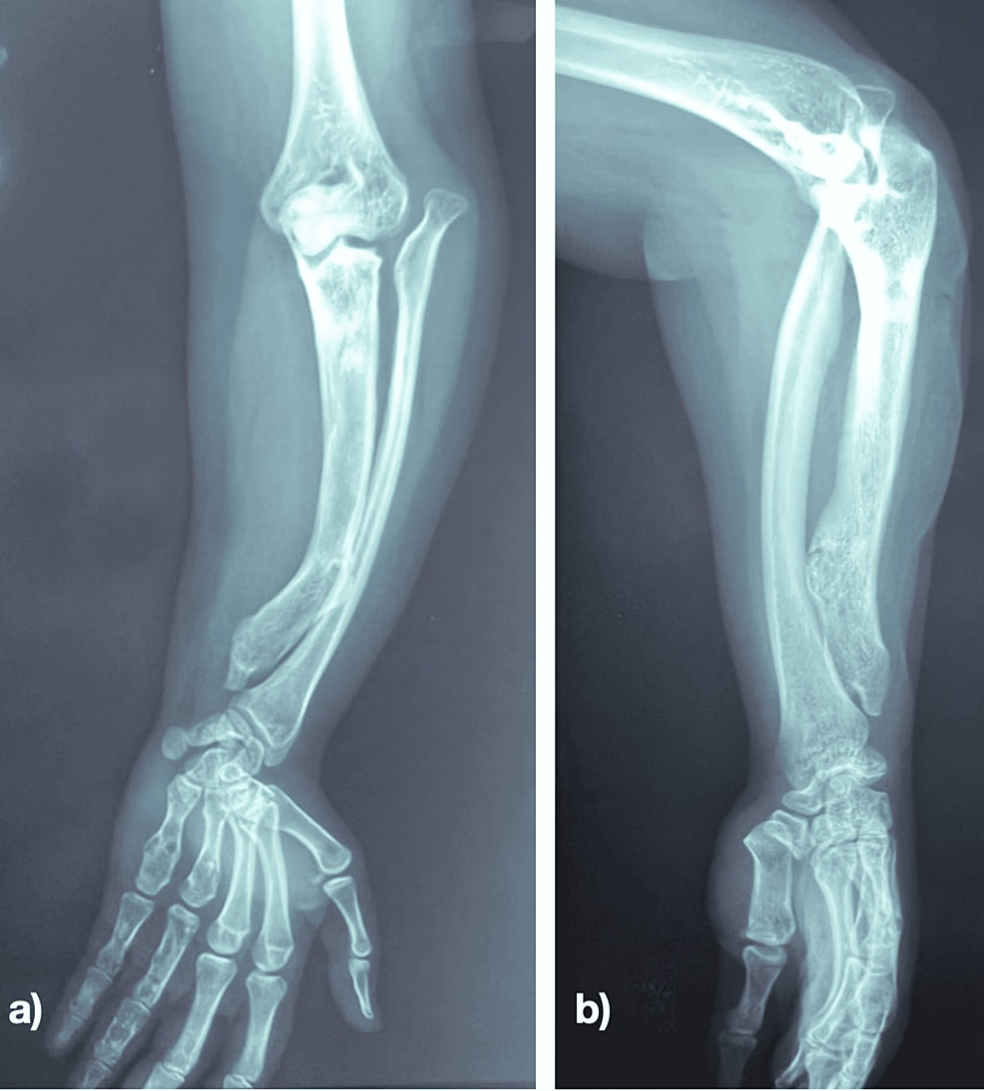
X-ray of the forearm before surgery: a) Anteroposterior X-ray of the forearm b) lateral X-ray of the forearm

The X-ray found a shortening of the ulna with a superior and lateral dislocation of the radial head, as well as a curved radial and ulnar shaft (Figure 2).

**Figure 3:**
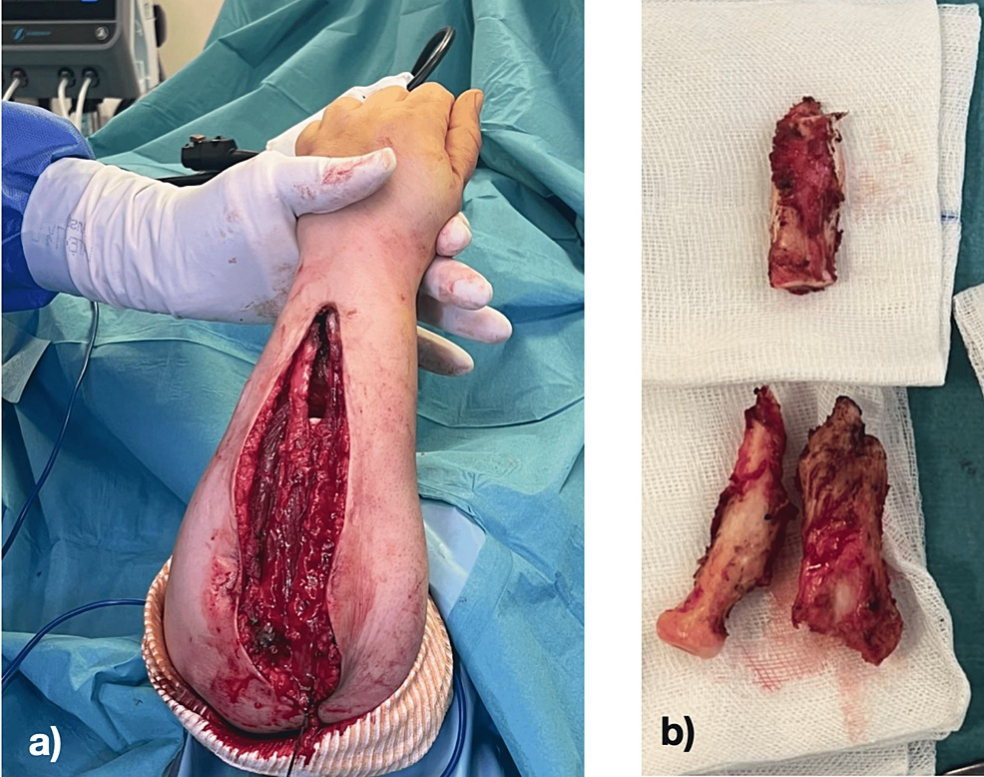
a) An intraoperative view of the approach b) The removed parts of ulna and radius

We chose a modified single bone forearm technique, via an extended ulnar approach and passing through the old scars, which were removed, as well as a hook on the lateral face of the elbow to reach the proximal end of the radius by the same approach (Figure 3).

We uninserted the brachial biceps tendon and placed it on a marker wire. We excised the radial head with a saw motor. We noted the presence of the motor branch of the radial nerve, driven back by the radial head, which was carefully dissected and reclined.

**Figure 4:**
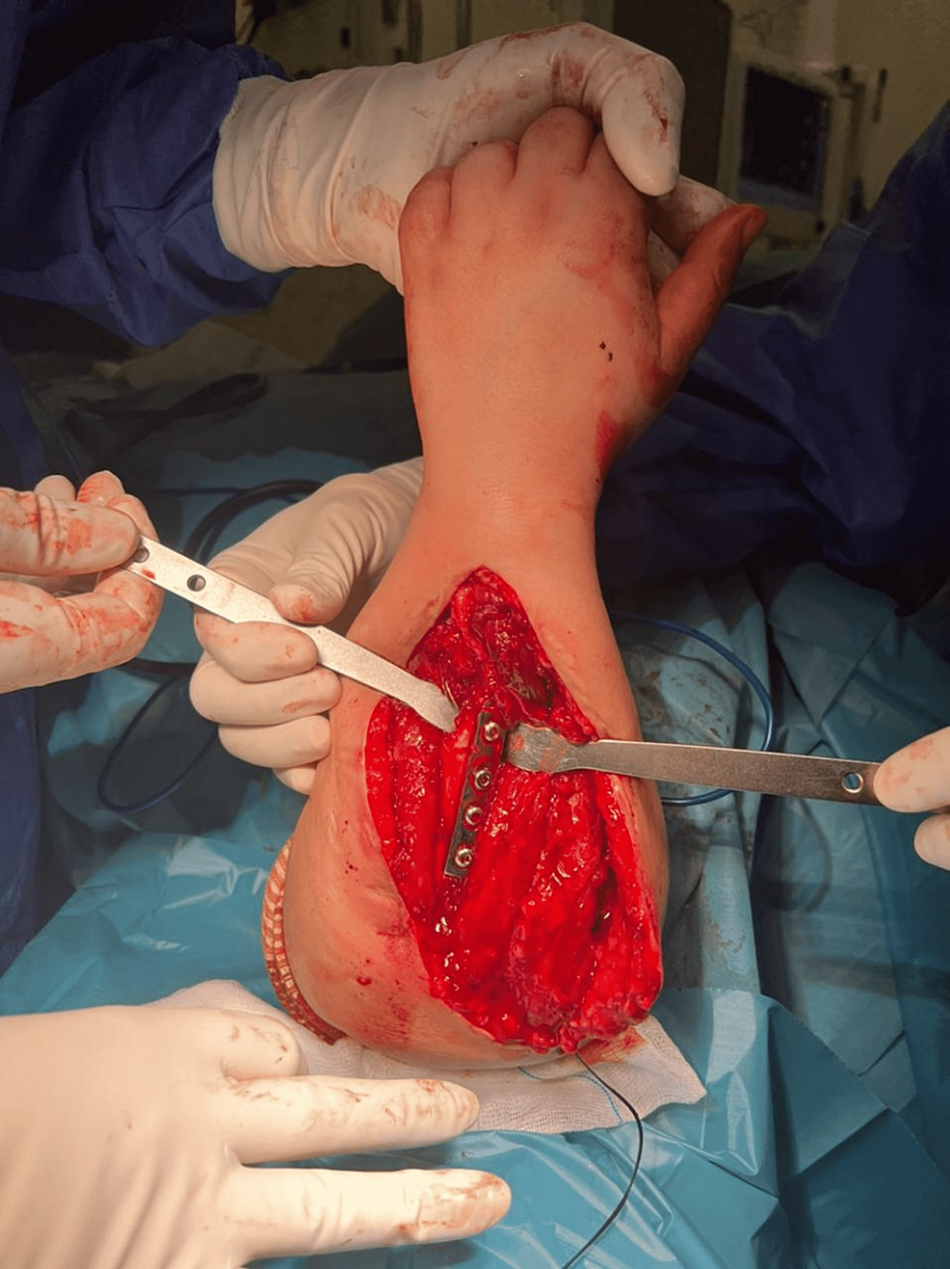
An intraoperative view showing the fixation of the ulna to the radius by a plate

**Figure 5:**
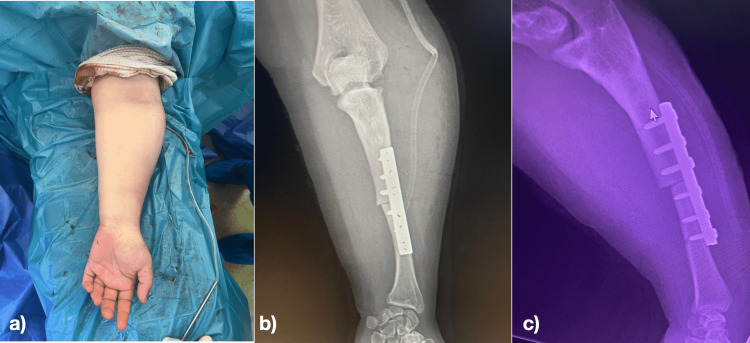
a) Immediate postoperative view of the forearm b) frontal Immediate postoperative X-ray c) lateral immediate postoperative X-ray

**Figure 6:**
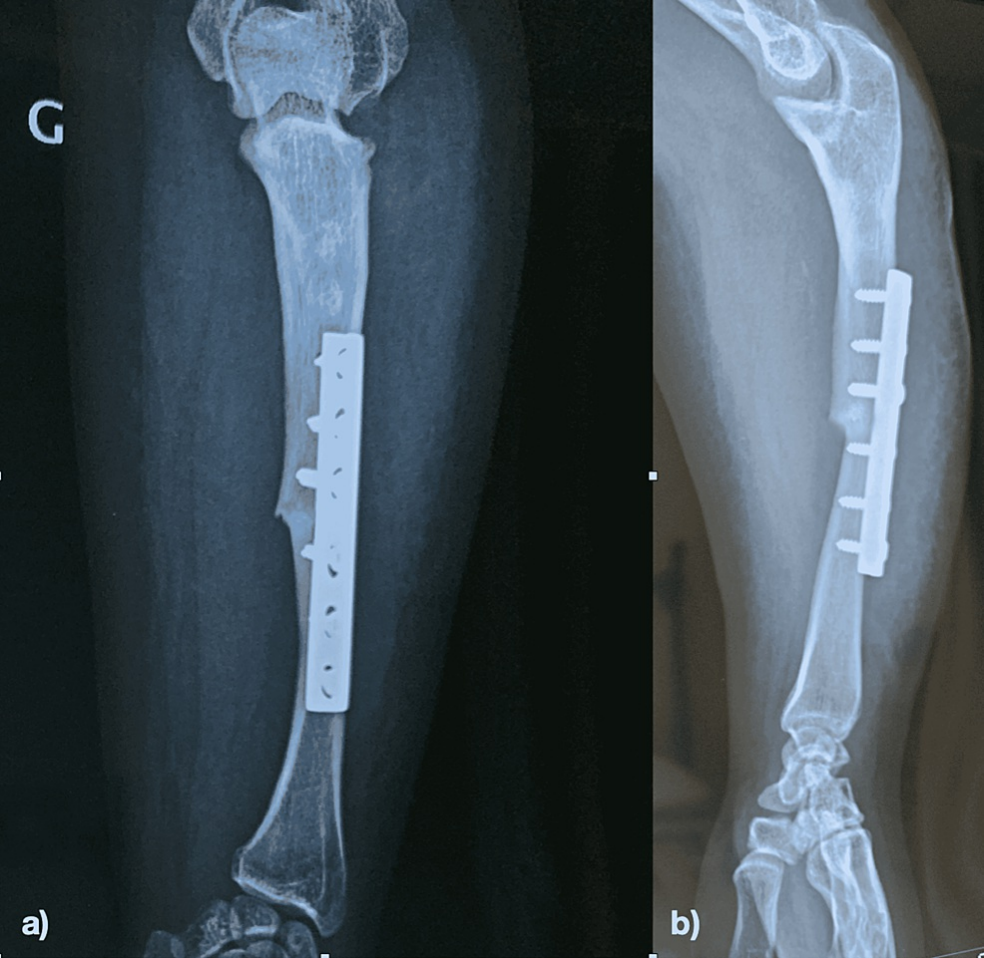
X-ray of the forearm after 4 months: a) Anteroposterior X-ray of the forearm. b) Lateral X-ray of the forearm

**Figure 7:**
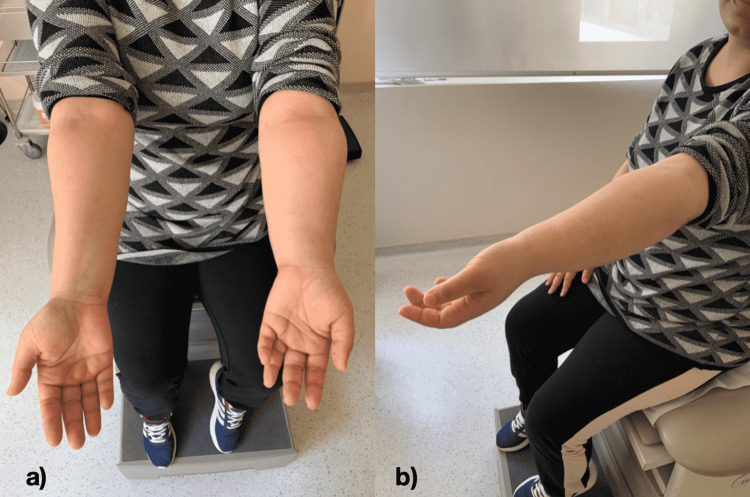
a) Realignment of the forearm. b) Full extension of the elbow

We excised the distal end of the ulna, then we corrected the axis to the radius by an oblique osteotomy and osteosynthesis of the remaining distal end of the radius and the proximal end of the ulna in slight pronation with a special radius plate. We reinserted the biceps tendon on the ulna (Figure 4). The immediate postoperative result found a normo-axis forearm, without neurological or vascular damages (Figure 5). The consolidation was obtained in four months (Figure 6). At 5 months, the patient recovered an elbow flexion at 110° and a full extension. The wrist flexion was at 45° and the extension at 50°. The radial inclination was at 20° and that of the ulnar was at 25° (Figure 7).

The pain score was 2/10 at three months after surgery, and the Quick dash score, at 45 post-operative days, was at 20,45/100.

## Discussion

Hereditary multiple exostoses disease is a fairly common benign tumor. It most often sits at the distal end of the ulna, causing more or less significant deformation and functional impotence [4,6,7].

Until now, no consensus has been established for its initial management [7]. However, the basic principle of treatment in childhood consists of resection of the exostosis associated with lengthening of the ulna, to avoid or reduce the dislocation of the radial head [4,8].

Despite adequate care, the persistence of various deformations in adulthood has motivated practitioners to find classifications in order to categorize these deformations. The two most reproducible classifications are those of MASADA and Jo [9]. In our case, the patient presented a MASADA 2B characterized by a deformation of the left forearm on a shortened ulna with a dislocation of the proximal radial-ulnar joint.

The single bone forearm technique has been described, not only for the treatment of hereditary multiple exostoses disease, but also for serious trauma or tumors with massive loss of bone [1-3]. The technique generally consists of an osteotomy of the radius as well as the ulna, then fixing the radius to the ulna with or without resection of part of one or both bones of the forearm [3,10].

Other different techniques were described using a vascularized fibular graft [11], a free fibula flap [12], a different fixation [13], or adding a fusion of the distal radioulnar joint [14].

In our case, there were multiple challenges. The main ones were a multi-operated forearm, and an aesthetic requirement of the patient who was embarrassed by the shape and the old scars, imposing on us a limitation of the approaches.

To our knowledge, no similar technique of single-bone forearm has been described in the literature. Generally, the technique consists of a partial resection of the bones of the forearm with parallel osteosynthesis of the radius at the ulna creating a synostosis [2]. In our case, given the discomfort induced by the significant dislocation of the proximal radial-ulnar joint, the obvious deformation and a shortened ulna, we had to remove the entire proximal half of the radius and the distal half of the ulna. Then, we fixed, end to end, the ulna to the radius, keeping only one axis of the forearm made of the ulna proximally and the radius distally.

Our modified single bone technique, as with other single bone techniques, does not aim at improving the wrist function. This technique helps to preserve the forearm, to realign it, and to maintain the functions of the elbow and wrist.

The most described complications of single bone forearm procedure are pain, complications related to soft tissue secondary to the previous injury, and infections [15].

In our case, there was no infection. The consolidation was obtained in four months with a normo-axis forearm. The elbow was stable without significant loss of range of motion. The pain rate was at 2/10 three months after surgery. This is in line with a previous study [16].

## Conclusions

The one-bone forearm remains a salvage technique for massive loss of bone of the forearm or large deformities due to congenital malformations. Keeping only one axis made of the proximal end of the ulna and the distal end of the radius did not show a limitation of motion or instability, neither of the elbow, nor of the wrist. This technique could allow the excision of massive bone while keeping only a part of the ulna and the radius, with functional and aesthetic forearm preservation.
